# ASO Author Reflections: Impact of Historical Redlining and Neighborhood Trajectories on Gastrointestinal Cancer Care

**DOI:** 10.1245/s10434-025-17880-y

**Published:** 2025-07-20

**Authors:** Mujtaba Khalil, Timothy M. Pawlik

**Affiliations:** Department of Surgery, Wexner Medical Center and James Comprehensive Cancer Center, The Ohio State University, Columbus, OH USA

## Past

Socioeconomic and racial disparities in cancer care have long been recognized; to date, most studies have examined neighborhood conditions using either historical or current indicators in isolation.^1^ Residential redlining—originating in the 1930s—systematically excluded minority populations from homeownership and investment, shaping patterns of disadvantage that persist today.^1^ More recently, indices such as the Social Vulnerability Index (SVI) have been used to assess current socioeconomic stressors at the community level.^2^ The interaction of past discriminatory policies and present-day social vulnerability affects cancer care remains not well examined, however. The current study sought to integrate historical redlining maps with contemporary SVI data to better understand how neighborhood trajectories influence gastrointestinal cancer diagnosis, treatment, and outcomes.^3^

## Present

Among 15,118 individuals, 30.5% (*n* = 4608) resided in advantaged stable neighborhoods, 44.5% (*n* = 6727) in disadvantaged reduced neighborhoods, 2.96% (*n* = 448) in advantaged reduced neighborhoods, and 22.1% (*n* = 3,335) in disadvantaged stable neighborhoods. Individuals living in disadvantaged stable neighborhoods were less likely to undergo surgery (55.8% vs. 59.2%), receive chemotherapy (56.7% vs. 60.3%), and achieve a textbook outcome following surgery (41.3% vs. 51.3%) (all *p* < 0.001). On multivariable analyses individuals living in disadvantaged stable neighborhoods had higher odds of being diagnosed at an advanced stage (OR 1.29, 95% CI 1.18–2.42), lower odds of receiving chemotherapy (OR 0.67, 95% CI 0.58–0.76), and achieving a TO (OR 0.68, 95% CI 0.59–0.77).

## Future

Structural inequities rooted in historical policy continue to shape cancer care and postoperative outcomes.^1^ The findings of current study underscore the need for a multidimensional approach to health equity—one that addresses not only current barriers such as insurance and transportation but also the enduring effects of structural racism.^3^ Policy reforms should focus on expanding access to cancer screening, strengthening support for safety-net hospitals, and funding community-based outreach programs.^4,5^ Improving screening rates in vulnerable neighborhoods will require investments in mobile screening units, culturally sensitive education, and partnerships with trusted local organizations.^6^ Equally important is the need to enhance access to timely and high-quality cancer centers.^4^ This includes expanding Medicaid coverage, increasing transportation infrastructure for medical visits, and supporting patient navigation services that can help individuals overcome logistical and administrative hurdles.^5^ This can be achieved through workforce development, better perioperative support systems, and incentivizing adherence to evidence-based clinical guidelines across institutions.^1^
